# IONPs combined with cytarabine downregulated IFITM3 expression to inhibit acute myeloid leukemia

**DOI:** 10.3389/fonc.2025.1515956

**Published:** 2025-09-03

**Authors:** Jun Dou, Feng Mei, Hui Xu, Xue Rui, Xueyang Bao, Kena Liu, Fengsu Zhao

**Affiliations:** ^1^ Academic Affairs Office, Xinjiang Second Medical College, Karamay, Xinjiang Ugur Autonomous Region, China; ^2^ Department of Pathogenic Biology and Immunology, School of Medicine, Southeast University, Nanjing, Jiangsu, China; ^3^ Department of Management, Western Yunnan University of Applied Science, Dali, Yunnan, China; ^4^ Department of Pathogenic Biology and Immunology, Jiangsu Vocational College of Medicine, Yancheng, Jiangsu, China; ^5^ Department of Obstetrics and Gynecology, Yangzhou University Affiliated Hospital, Yangzhou, Jiangsu, China; ^6^ Affiliated Pediatric Hospital of Nanjing Medical University, Nanjing, Jiangsu, China

**Keywords:** acute myeloid leukemia, interferon-induced transmembrane protein 3, iron oxide nanoparticles, cytarabine, reactive oxygen species, acute myeloid leukemia-bearing mice

## Abstract

**Background and objective:**

Acute myeloid leukemia (AML) is the most prevalent acute leukemia in adults. While conventional therapies can induce remission disease frequently relapses with fatal outcomes. This study aimed to explore the inhibitory effect of iron oxide nanoparticles (IONPs) combined with cytarabine (Ara-C) on AML by modulating the interferon-induced transmembrane protein 3 (IFITM3) expression of in KG-1a cells and AML-bearing mice.

**Methods:**

A lentiviral vector targeting *IFITM3* (LV-shIFITM3) was used to transduce human AML KG-1a cells, and the biological effects of IFITM3 knockdown were assessed. NOD/SCID mice were engrafted with LV-shIFITM3-KG1a cells and their survival status as well as abnormal cell population were monitored.

**Results:**

Compared to control cells, IFITM3 expression level in the LV-shIFITM3 transduced KG-1a cells exhibited reduced IFITM3 expression, leading to suppressed proliferation, impaired clonogenicity, cell cycle arrest, and increased apoptosis. The combination of IONPs and Ara-C further diminished IFITM3 level, inhibited KG-1a proliferation, and induced apoptosis while elevating reactive oxygen species (ROS) production. *In vivo*, IONPs plus Ara-C treatment reduced immature granulocytes in peripheral blood and bone marrow, downregulated AML-associated markers (clustered differentiation(CD)33 and CD11b), and ameliorated disease progression in AML-bearing mice compared to controls.

**Conclusion:**

IFITM3 knockdown in KG-1a cells inhibited proliferation and promoted apoptosis. The combination of IONPs and Ara-C may represent a promising therapeutic strategy for AML by suppressing IFITM3 expression, enhancing ROS levels, and inducing apoptosis. These findings suggest IFITM3 as a potential molecular target and highlight the synergistic efficacy of IONPs and Ara-C in AML treatment.

## Introduction

Acute myeloid leukemia (AML) is the most common acute leukemia in adults, comprising approximately 80% of adult leukemia patients. This highly heterogeneous disease driven by transcriptional dysregulation, resulting in impaired differentiation, uncontrolled proliferation of immature myeloid blasts, bone marrow failure, and eventual infiltration of extramedullary organs, such as the liver, lungs, spleen, and lymph nodes. The current standard of care- combinating cytosine arabinoside (Ara-C) with anthracyclines based chemotherapy followed by allogeneic stem cell transplantation in eligible patients-induces remission in a majority of cases. However, disease relapse remains a significant challenge, leading to poor long-term survival, particularly in high-risk subgroups, and this is because AML are resistant to chemotherapy (e.g., cytarabine/Ara-C), leading to relapse (1, 2). Moreover, older AML patients, who represent the majority of cases, often exhibit poor tolerance to intensive therapies, underscoring the urgent need for novel treatment strategies (3, 4).

The interferon-induced transmembrane protein 3 (IFITM3), encoded on chromosome 11, is a key member of the *IFITM* family with roles in cell differentiation, apoptosis, inflammation, and immune modulation (5, 6).While IFITM3 overexpression has been implicated in the progression of multiple solid tumors, such as promoting proliferation, metastasis, invasion, and epithelial-mesenchymal transition (EMT) (7–9),its role in AML remains poorly understood.

Iron oxide nanoparticles (IONPs) represent an emerging class of nanozymes that exhibit intrinsic peroxidase-like activity, mimicking horseradish peroxidase (HRP). Under acidic conditions, IONPs catalyze the conversion of hydrogen peroxide (H_2_O_2_) into hydroxyl radicals (OH), elevating intracellular reactive oxygen species (ROS) levels and inducing oxidative stress-mediated cell death (10, 11). Previous study has revealed that Iron oxide nanoparticles (IONPs) may disrupt AML by increasing ROS via Fenton reaction, while sparing normal hematopoietic stem cells (HSCs). This provides a theoretical basis for the treatment of AML.

To explore a novel therapeutic approach for AML, we investigated the synergistic efficacy of IONPs combined with cytarabine by targeting IFITM3 in the human AML cell line KG-1a. Using lentiviral vector-delivered small hairpin RNA (shRNA) to silence IFITM3 (shIFITM3), we evaluated the synergistic effects of IONPs and Ara-C *in vitro* and *in vivo*. Additionally, we assessed the treatment outcomes and underlying mechanisms in AML-bearing mice.

## Materials and methods

### Cell culture

The human AML cell line KG1a for the study was generously provided by the laboratory of Professor Xu Haiyan, Academy of Basic Medical Sciences, Beijing Academy of Medical Sciences. The cells were cultured in a special cell incubator with 5% CO_2_ at 37°C with saturated humidity using IMDM containing 10% fetal bovine serum(GIBCO, USA) and 1×Penicillin and streptomycin mixture. The cells used in the experiment were in the logarithmic growth stage.

### Liposomal transfection and lentivirus infection of KG-1a cells

According to the Lipofectamine 2000 manufacturer’s specifications (Invitrogen, USA), IFITM3 empty vector and IFITM3 silenced plasmid (sh-IFITM3 (Forward: 5’-CCAUUCUGCUCAU CGUCAUTT-3 ‘; Reverse: 5’-AUGACGAUGAGCAGAA UGGTT-3’) were transfected into the KG-1a cells. Lentiviral vector was purchased from Shanghai Jikai Biotechnology Co., LTD. The recombinant lentivirus was constructed according to sequences that could effectively silence IFITM3 expression. The constructed lentivirus was used to infect the KG-1a cells by following the instructions from Geke Co. The positive cells (LV-shIFITM3 cells) were screened using purinomycin.

### KG-1a cell proliferation assay

The stable infected cells screened by 3 μg/ml purinomycin were prepared into single-cell suspension using IMDM medium containing 10% FBS, and the cell concentration was adjusted to 5×10^4^/ml. 100μl (5000 cells) were inoculated into 96-well plates, with 18 holes in each experimental group and 18 control holes, cultured at 37°C in 5% CO_2_ incubator. The 3-well cells were harvested from each group at 0h, 24h, 48h, 72h and 96h and 10μl CCK8 (Cell Counting Kit-8) solution was added to each cell well, incubated in the incubator-CO_2_ for 4h (lighting was avoided during this step). The absorbance (OD) value was measured at 450 nm by an enzyme-labeled apparatus with the blank control of cell culture medium and CCK8 solution only. The CCK-8 assay is a widely used colorimetric method to measure cell proliferation, viability, and cytotoxicity. Cell proliferation rates (taking 24h cell culture as an example) were calculated according to the following formula (12).: Cell proliferation rate (%) = (24h OD value -0h OD value)/(0h OD value - OD value of blank control group) ×100.

### Apoptosis and ROS levels of KG-1a cells were detected by FCM

The KG-1a cells in the logarithmic growth phase, scrambled-shRNA and sh-IFITM3-KG1a cells were adjusted to 1×10^6^/ml, respectively, treated with Ara-C (Ara-C 0.4μM) at the same time, and placed in a cell incubator. After 24 hours of culture, the cells were collected and placed into the tubes. Another 2×10^6^ KG1a cells in normal culture were divided into three parts and used as voltage regulation and Annexin V and PI monostaining tubes, respectively. 1ml of 1× apoptosis kit buffer was added to each tube, and the cell apoptosis was detected and analyzed by flow cytometry (FCM) according to apoptosis detection procedures. In addition, the KG-1a cells were treated with PBS control, Ara-C (Ara-C 0.4μM (Shanghai McLean Biotechnology Co., LTD), IONPs (IONPs 150μg/ml, provided by Prof. Yu Zhang, School of Biological Science and Medical Engineering, Southeast University), Ara-C+IONPs (Ara-C 0.4μM; IONPs 150μg/ml), respectively, and then an appropriate amount of 10 μM DCFH-DA was added to each tube to adjust the cell concentration to 2×10^6^/ml. Finally, each tube was suspended with 500μl PBS before FCM was used to detect the ROS levels according to ROS detection procedures. The biomarkers of CD11b,CD33 and CD123 were analyzed by FCM using CD11b-FITC anti-human antibody,CD33-PE anti-human antibody and CD123-PE anti-human antibody(Biolegend Co.).

### KG-1a cell cloning assay in soft agar

Preparation of cell suspension: the 5×10^4^/mL KG1a cells, the scrambled-shRNA and the sh-IFITM3-KG1a cells were prepared into single-cell suspension with IMDM culture medium containing 10% FBS, adjusted to a concentration of 2×10^3^/mL, and placed into a cell incubator for heat preservation. The preparation of the soft agar, cell counting and the subsequent steps were performed according to the previous protocol (13).

The clone formation rate (%) = (number of cell clones containing more than 50 cells/inoculated cells) ×100%.

### Quantitative reverse transcriptase-PCR and Western blot assays

The 5 ×10^4^ per ml KG1a cells in the logarithmic phase were treated with PBS control, Ara-C, IONPs (IONPs 150μg/ml), and IONPs plus Ara-C (Ara-C 0.4μM; IONPs 150μg/ml), respectively, and then placed in a cell incubator. After 24h of the treatment, the KG1a cells were collected from each group and placed in 1.5ml EP tubes. Next, another batch of the 5×10^4^/ml KG-1a cells, the scrambled -shRNA, and the sh-IFITM3-KG1a cells were taken; the subsequent total RNA was extracted and RT-qPCR was performed following the description in the published papers (14, 15). The sequences of the primers are as follows: the IFITM3 forward, 5′- TCACACTGTCCAAAC CTTCTTCT -3′; reverse, 5′- TTGAACAGGGACCA GACGAC-3′; the Caspase 3 forward, 5′- CCTGGTTCATCCAGTCGCTT3′; reverse, 5′- TC TGTTGCCACCTTTCGGTT3′; the GAPDH forward, 5′- CGGAGTCAACGGATTTGGT CGTAT-3′; reverse, 5′- AGCCTTCTCC ATGGTGGTGAAGAC-3′. The qRT-PCR analysis was performed on an ABI step one plus real-time system (Applied Biosystems).

The KG1a cells were treated in the same way as described above. Western blot was performed to detect the expression levels of related proteins reported previously (16). Briefly, the proteins (10 μg/lane) were transferred onto a PVDF membrane blocked with 4% dry milk in Tris-buffered saline with Tween-20 for 1h at 20 °C. The membrane was then incubated with the rabbit anti-human GAPDH monoclonal antibody and the rabbit anti-human IFITM3 polyclonal antibody (Wuhan Sanying Biotechnology Co. LTD), respectively, for overnight at 4°C. Next, the membrane was rinsed with an antibody wash solution for 3 times, 5 min per wash, before the goat anti-rabbit secondary antibody was added. The subsequent steps were performed according to the Western Blot Kit’s protocol (Pierce Co.).

### *In vivo* AML experiments in mice

Female non-obese diabetic/severe combined immune deficiency(NOD/SCID) mice, aged 6 weeks, weighing (16.88 ± 0.51) g, were purchased from Beijing Weitonglihua Laboratory Animal Technology company (Co.), LTD., license No. SCXK (Beijing) 2016-0006. The acquired mice were housed in a pathogen-free facility. This study followed all relevant ethical regulations on animal research according to the protocols approved by the Animal Use Committee at Southeast University. The mice were fed adaptively for 3 days at a pathogen- free facility and randomly divided into six groups of 3 mice each: a scrambled-shRNA group, a sh-IFITM3-KG1a cell group, and four groups of uninfected KG-1a cells. The cell concentration was adjusted to 5×10^7^/ml and 0.1ml was inoculated into the tail vein of each mouse. Starting from the first day of the cell inoculation, blood was collected from the posterior orbital venous plexus of each mouse once every 7 days and sent for blood routine analysis in a lab. The changes in the disease status of the mice were observed and recorded every day. Starting from day 15 of the cell inoculation, the mice inoculated with the KG-1a cells were treated with PBS, Ara-C, IONPs and Ara-C+IONPs, (Ara-C 50mg/kg/each/time), respectively; the mice were treated with IONPs (10mg/kg/each/time) once every 3 days for a total of 10 times. The mice were killed routinely after the 30-day treatment, and justify the doses and timing of IONPs and Ara-C used in both *in vitro* and *in vivo* experiments with reference to prior study (17). The harvested livers and spleens were dissected and soaked in 4% paraformaldehyde, and the smears of peripheral blood and bone marrow were taken for observation. The effect of treatment for the AML model mice was analyzed by combining the observation indexes. The experiment was repeated twice.

### Statistical analysis

All experimental results were expressed as mean ± standard deviation and statistically analyzed using PraphPad Prism 5.0. Independent sample *t* tests were used for between-group comparisons, and *P*< 0.05 was considered statistically significant.

## Results

### IFITM3-specific shRNA effectively silenced IFITM3 expression in KG-1a cells

**Figure 1:**
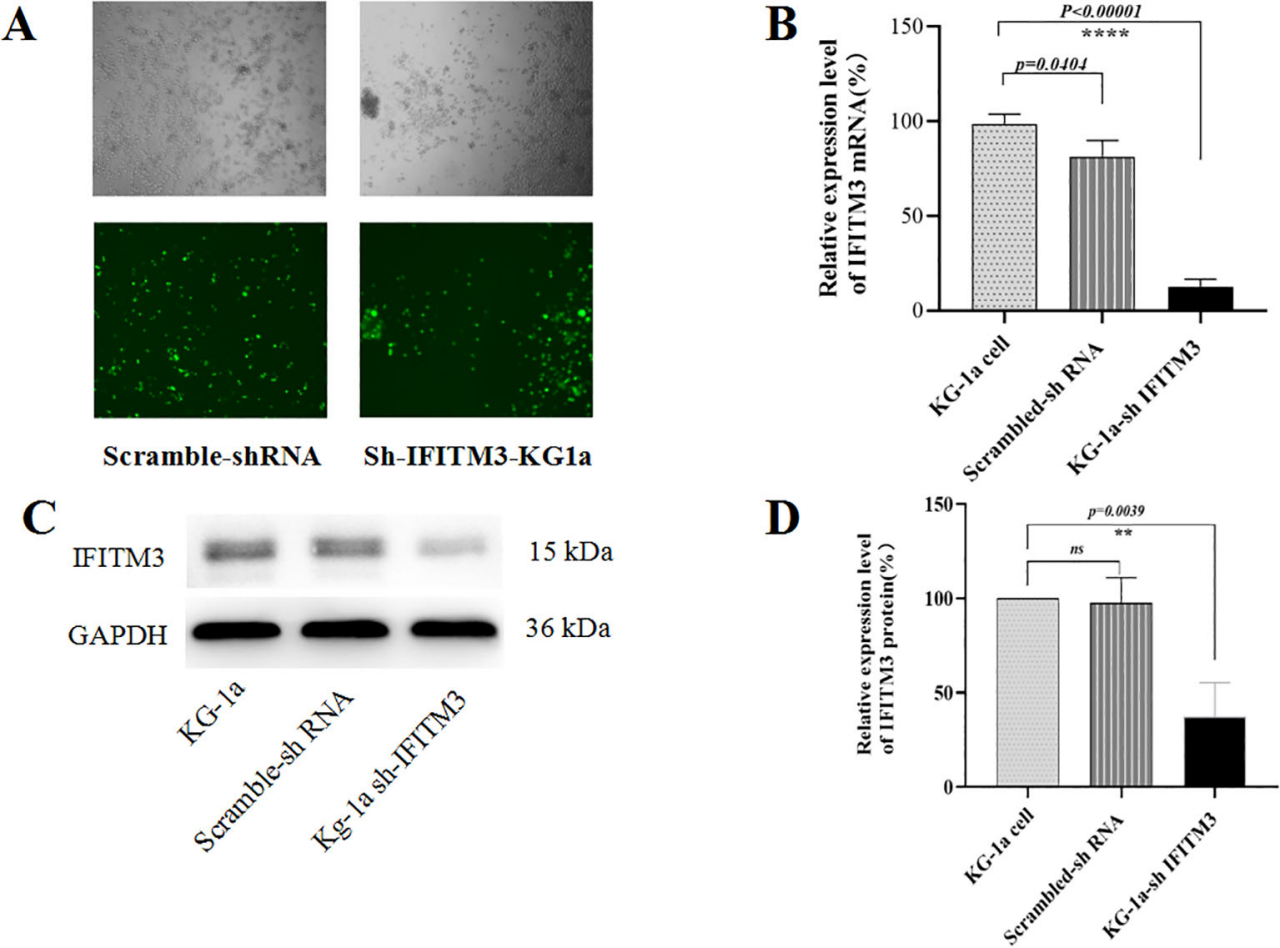
Analyzing the knockdown of IFITM3 efficiency in LV-shIFITM3 KG-1a cells. **(A)** The efficiency of lentivirus infection of the KG-1a cells was observed under a conventional fluorescent microscope. **(B)** The IFITM3 mRNA expression in the KG-1a cells was detected by qRT-PCR. **(C)** The expression of the IFITM3 protein in the KG-1a cells was detected by Western blot. **(D)** The results from the semi-quantitative analysis of protein expression levels. ****P<0.00001. ns, no statistical significance.

Since the lentiviral recombinant vector carried the enhanced green fluorescent protein (EGFP) gene, transfection efficiency was confirmed by observing EGFP fluorescence under a microscope. As shown in **Figure 1A**, over 90% of cells were EGFP-positive after a 72-hour puromycin selection. qRT-PCR analysis revealed a significant reduction in IFITM3 mRNA levels in Lv-shIFITM3-KG1a cells compared to uninfected and scrambled control cells (*p < 0.05*, **Figure 1B**). Consistent with this, Western blotting demonstrated markedly decreased IFITM3 protein expression in Lv-shIFITM3-KG1a cells (**Figure 1C**). Densitometric analysis further confirmed that IFITM3 expression was significantly lower in the knockdown cells than in the control groups (**Figure 1D**). Collectively, these results validate the successful generation of KG-1a cells with stable IFITM3 downregulation.

### IFITM3 affected the biological properties of KG-1a cells

**Figure 2:**
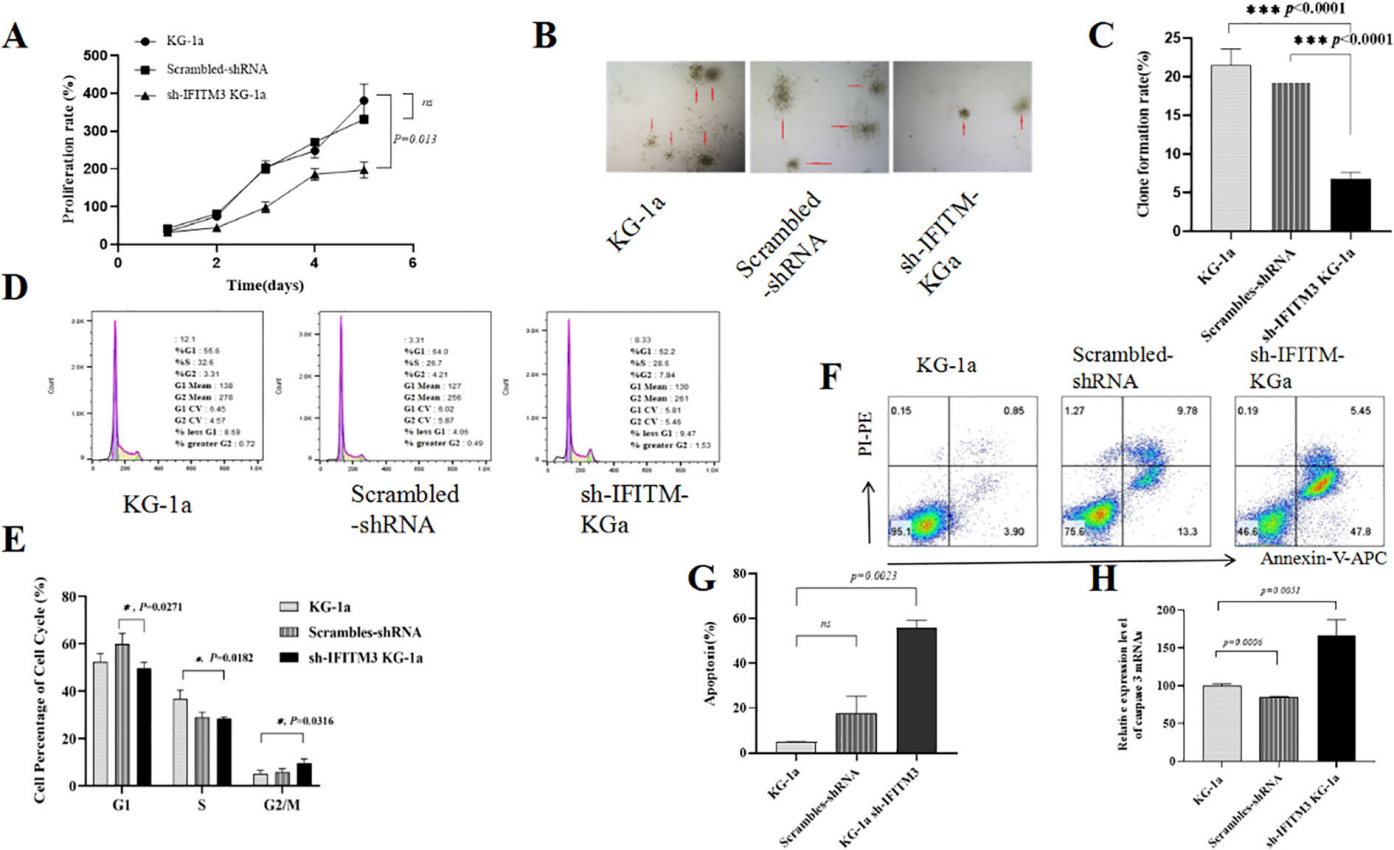
Down-regulation of IFITM3 changed the biological properties of the KG-1a cells. **(A)** The effect of down-regulated IFITM3 on the proliferation ability of KG-1a cells as detected by CCK8 assay. **(B)** The colony formations of various KG-1a cells detected by soft agar cloning assay (50×); **(C)** Comparisons of the colony formations; **(D)** The FCM analysis results of the KG-1a cell cycle changes; **(E)** Comparisons of the cell cycles; **(F)** The effects of Ara-C (0.4μM) on the apoptosis of various KG-1a cells analyzed by FCM (24h). **(G)** The FCM results; **(H)** The mRNA expression level of caspase 3 in various KG-1a cells as detected by qRT-PCR. *P < 0.05, ***P < 0.001. ns, no statistical significance.

The CCK8 experiment showed a significantly reduced proliferation rate in the Lv-shIFITM3- KG1a cells compared with the KG1a and the scrambled cells (**Figure 2A**). The colony formation results indicated that the down-regulated IFITM3 expression in the KG-1a cells significantly inhibited the clonogenesis (**Figures 2B, C**). The cell cycle experiment displayed that the down-regulated IFITM3 expression blocked the KG-1a cells in the G2/M phase (**Figures 2D, E**). Flow cytometry (FCM) analysis of Ara-C-resistant cells demonstrated that down regulation of IFITM3 expression enhanced Ara-C-induced apoptosis in KG-1a cells (**Figures 2F, G**). The qRT-PCR results showed that the expression of the apoptosis-related molecule caspase 3 was significantly increased after the down-regulation of the IFITM3 expression (**Figure 2H**). These results suggested that the down-regulated IFITM3 expression inhibited the KG-1a proliferation and promoted further KG-1a apoptosis that was induced by chemotherapy drug Ara-C.

### Downregulation of IFITM3 inhibits NF-κB p65 phosphorylation and c-myc expression

**Figure 3:**
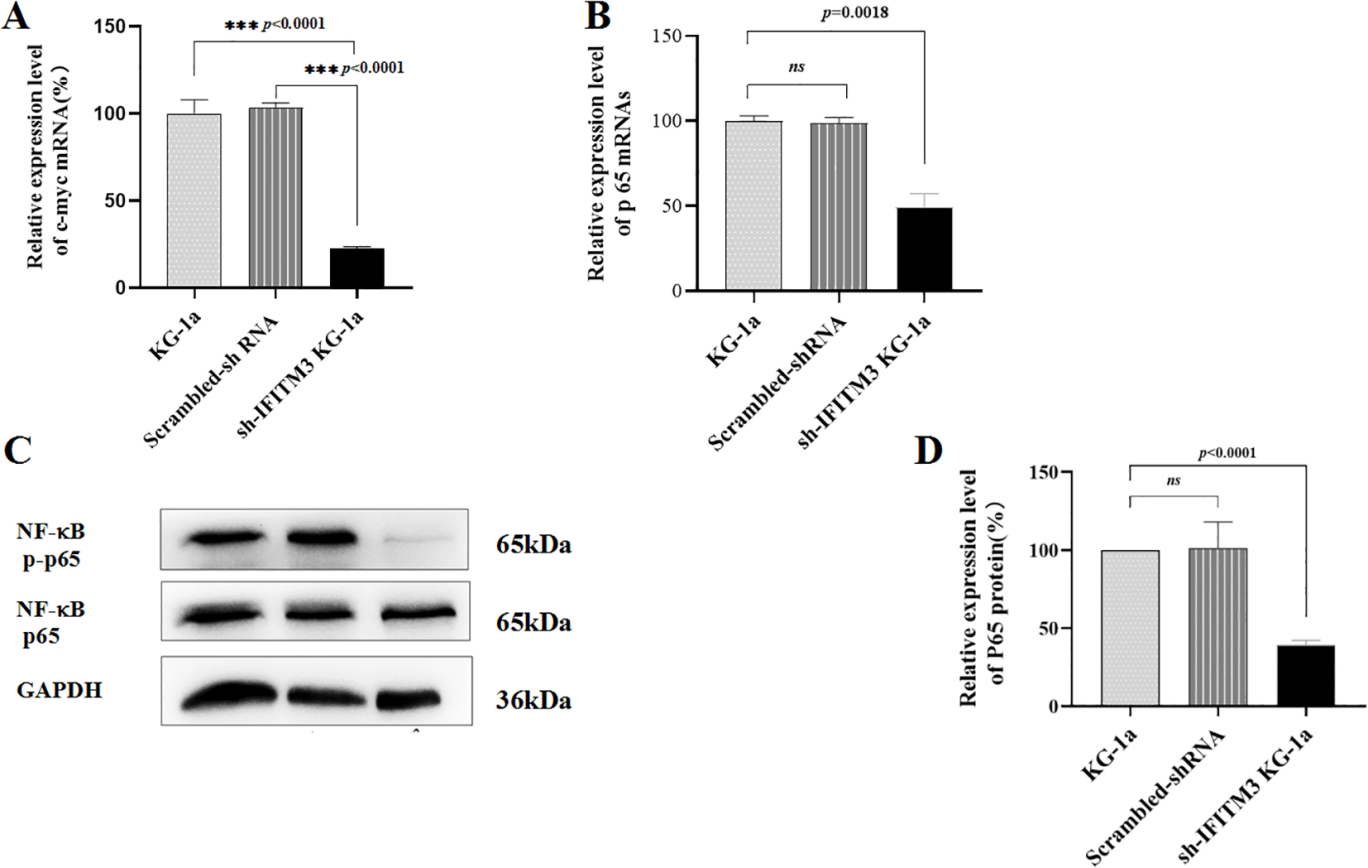
The expression levels of NF-κB p65 and c-myc. **(A, B)** The mRNA expression levels of c-myc and p65 in the KG-1a cells as detected by qRT-PCR. **(C)** The expression levels of p65 protein and the phosphorylation of NF-κB p65 in the KG-1a cells analyzed by Western blot. **(D)** The semi-quantitative analysis of the Western blot results. ns, no statistical significance.

As shown in **Figure 3A**, knockdown of IFITM3 significantly suppressed cell proliferation; however, the underlying mechanism by which IFITM3 regulates AML cell proliferation remains unclear. Previous studies have implicated the NF-κB signaling pathway in promoting tumor cell proliferation, while c-myc has been reported to correlate positively with IFITM3 expression in liver cancer cells though this relationship has not been established in AML. In this study, we investigated the effects of IFITM3 downregulation on NF-κB p65 in KG-1a cells using qRT-PCR and Western blot. **Figures 3A and B** demonstrate that the mRNA expression levels of c-myc and NF-κB p65 were significantly reduced in Lv-shIFITM3-KG1a cells compared to both control KG1a and scrambled cells. Furthermore, Western blot analysis revealed that IFITM3 knockdown markedly inhibited NF-κB p65 phosphorylation (**Figures 3B–D**). These findings suggest that IFITM3 silencing may impair KG-1a cell proliferation by suppressing the NF-κB p65 and c-myc pathways.

### IONPs inhibited IFITM3 expression and proliferation in KG-1a cells

In our previous study, we demonstrated that nanozyme-like IONPs modulate ROS levels in KG-1a cells by upregulating the pro-oxidative molecule gp91-phox and downregulating the antioxidative molecule superoxide dismutase (SOD) (17).

**Figure 4:**
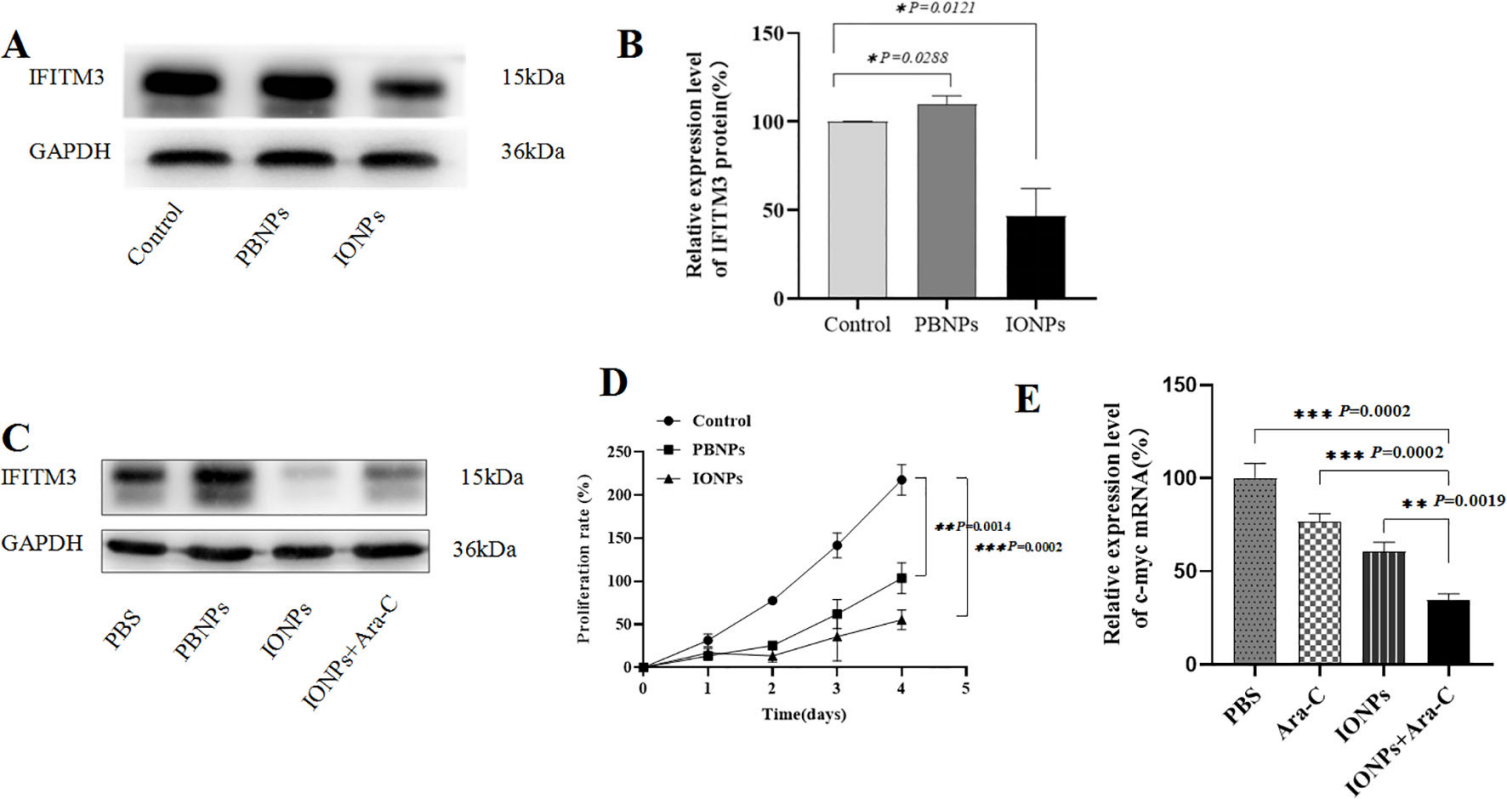
The IFITM3 expression and the proliferation of the KG-1a cells. **(A)** The IFITM3 expression as detected by Western blot after the KG-1a cells were treated with PBS, PBNPs and IONPs, respectively, for 72h; **(B)** The semi-quantitative analysis of the Western blot results; **(C)** The IFITM3 expression in the KG-1a cells as detected by Western blot after the cells were treated with PBS, PBNPs and IONPs, respectively, for 72h; **(D)** The proliferation of the KG-1a cells as detected by the CCK8 assay in the KG-1a cells that were treated with different drugs for 96h; **(E)** The qRT-PCR-detected expression level of c-myc in the KG-1a cells treated with different drugs for 24h. *P < 0.05, **P < 0.01, ***P < 0.001. ns, no statistical significance.

In this study, we investigated whether IONPs regulate IFITM3 expression and influence the biological properties of KG-1a cells. As shown in **Figures 4A, B**, IONPs significantly downregulated IFITM3 expression compared to Prussian blue nanoparticles (PBNPs) after 72 hours of treatment. Furthermore, when KG-1a cells were treated with IONPs alone or in combination with Ara-C for 72 hours, IONPs consistently suppressed IFITM3 expression, with a statistically significant difference compared to Ara-C treatment alone (**Figure 4C**).

Additionally, IONPs markedly inhibited KG-1a cell proliferation compared to PBNPs (**Figure 4D**). We hypothesized that this anti-proliferative effect might involve c-myc downregulation. Quantitative RT-PCR analysis supported this hypothesis, revealing that IONPs significantly reduced c-myc mRNA levels (**Figure 4E**), which may contribute to the observed suppression of KG-1a cell proliferation.

### IONPs and Ara-C induced cell apoptosis via ROS mediated pathway

**Figure 5:**
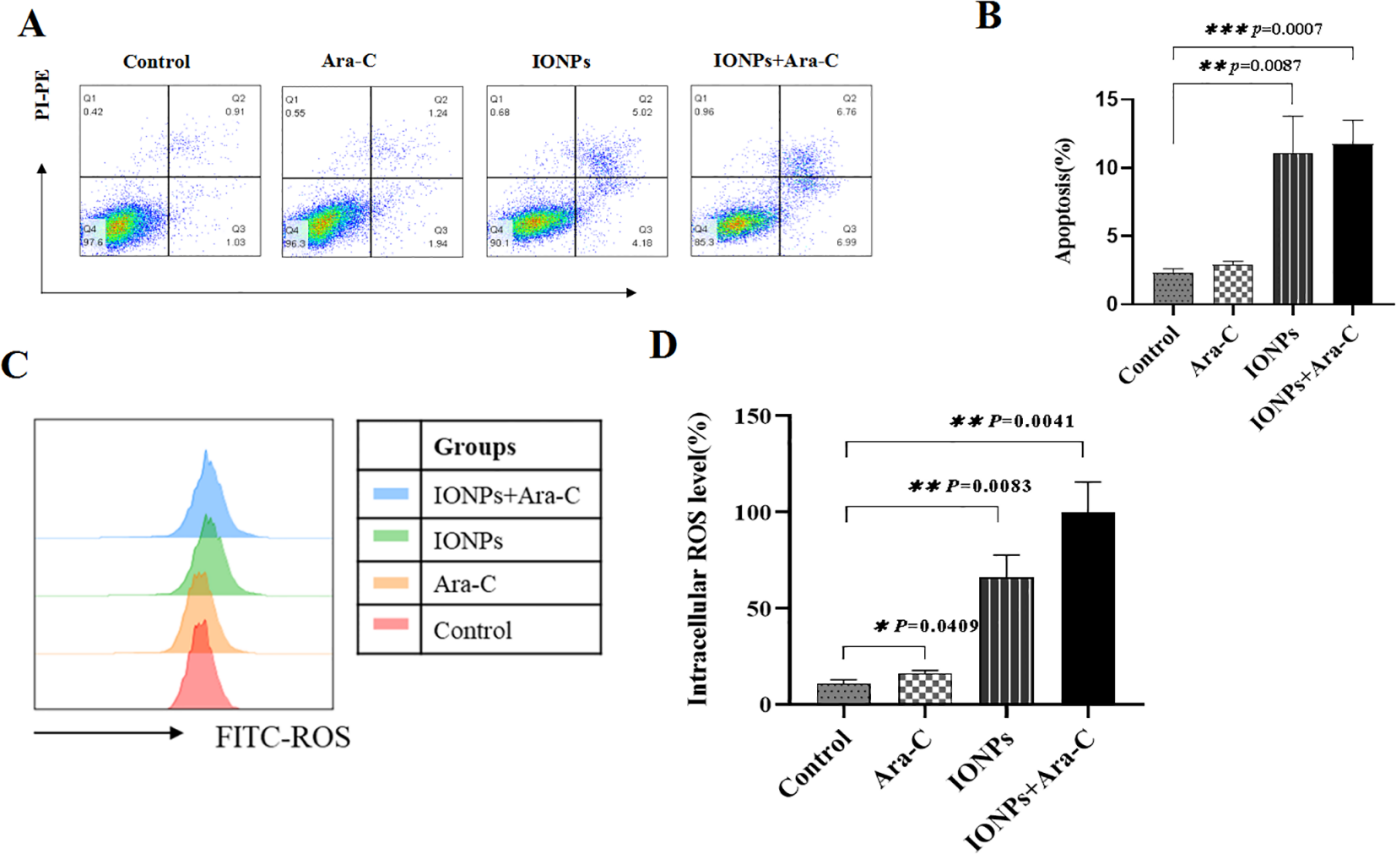
IONPs and Ara-C promote KG-1a apoptosis and ROS production. **(A)** KG-1a apoptosis as detected by FCM. **(B)** Analysis results of apoptosis; **(C)** Cell ROS levels analyzed by FCM; **(D)** Results of the cell ROS levels. *P < 0.05, **P < 0.01, ***P < 0.001. ns, no statistical significance.

The FCM analysis revealed that KG-1a cells treated with either Ara-C or IONPs exhibited significantly higher apoptosis rates compared to untreated controls. Notably, the combination of IONPs and Ara-C synergistically enhanced apoptosis (**Figures 5A, B**). To elucidate the underlying mechanism, intracellular ROS levels were measured. FCM results demonstrated that both Ara-C and IONPs monotherapies markedly increased ROS production in KG-1a cells relative to controls. Strikingly, the IONPs+Ara-C combination group showed the highest ROS accumulation among all treatment groups, with statistically significant differences (**Figures 5C, D**).

### Down-regulation of IFITM3 inhibits the leukemia cell proliferation in mice

**Figure 6:**
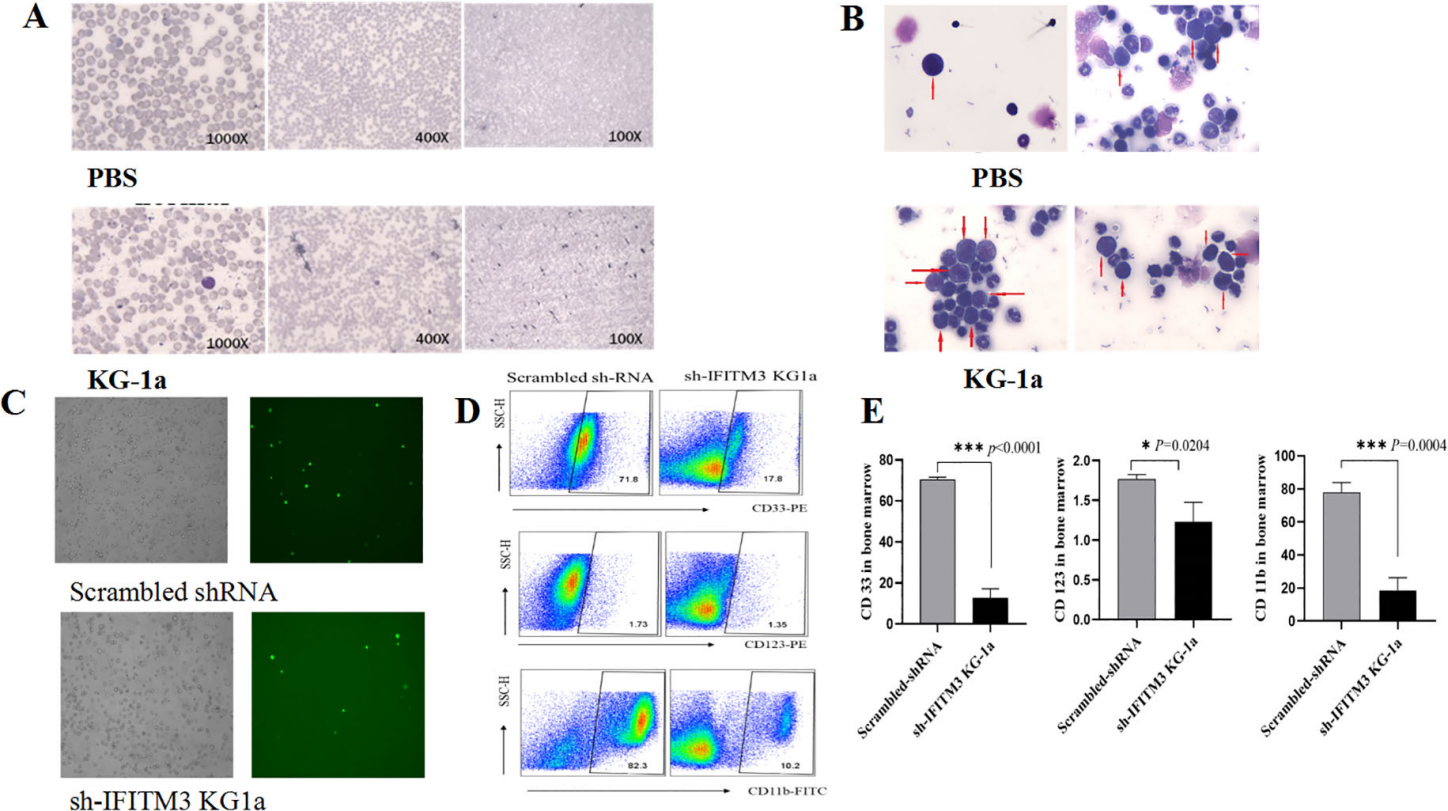
Impact of IFITM3 knockdown on disease progression in AML model mice. **(A)** Leukemia cells in peripheral blood and bone marrow; **(B)** With Reichsen-Giemsa staining; **(C)** Leukemia cells of bone marrow (left: under a light microscope; right: under a fluorescence microscope) in the AML-bearing mice injected with the Scrambled or the Lv-shIFITM3-KG1a cells; **(D)** The expression levels of CD33, CD123 and CD11b detected by FCM. **(E)** Statistical results of the CD33, CD123 and CD11b expression levels. The experiment was repeated twice. *P < 0.05, ***P < 0.001.

To investigate the role of IFITM3 downregulation in acute myeloid leukemia (AML), we first established an AML mouse model by intravenously injecting 5×10^6^ KG-1a cells into NOD/SCID mice via the tail vein. Thirty days post-injection, leukemia cells were detected in peripheral blood and bone marrow smears from AML-bearing mice, whereas no leukemic cells were observed in PBS-treated control mice (**Figure 6A**).

Bone marrow smears from AML-model mice exhibited a pronounced increase in primitive and immature cells, displaying characteristic leukemic features such as irregular morphology, condensed nuclear chromatin, hyperchromatic staining, and dysplastic nuclear-to-cytoplasmic ratios. In contrast, only minimal primitive cells were observed in PBS-treated controls (**Figure 6B**). These findings confirmed the successful establishment of the AML mouse model.

Next, 6 NOD/SCID mice were randomly divided into 2 groups of 3 mice each. Either the 5×10^6^ scrambled IFITM3-KG1a or the Lv-shIFITM3-KG1a cells were implanted into each mouse through its tail vein; the changes in the mice were observed and recorded daily. On Day 40, the mice injected with the scrambled cells were found to exhibit drowsiness, hypokinesis and hair loss; however, none was seen in the Lv-shIFITM3-KG1a mice. Since the lentiviral recombinant vector contains the green fluorescent protein gene EGFP, the IFITM3 expression in either the IFITM3-KG1a or the Lv-shIFITM3-KG-1a cells can be observed under a fluorescence microscope. **Figure 6C** shows that the fluorescence expression was significantly reduced in the Lv-shIFITM3-KG1a group compared with the scrambled group. The proliferation rate of the Lv-shIFITM3-KG1a cells in the model mice was significantly slowed down. To further verify this finding, FCM analyses were conducted and detected the expressions of the AML-related immunological markers, CD33, CD123, and CD11b, in the Lv-shIFITM3-KG1a cells. **Figures 6D, E** show that the expression levels of CD11b, CD33, and CD123 were decreased, with statistically significant differences in comparison to the scrambled cells.

### Therapeutic effect of IONPs combined with Ara-C on the AML model mice

**Figure 7:**
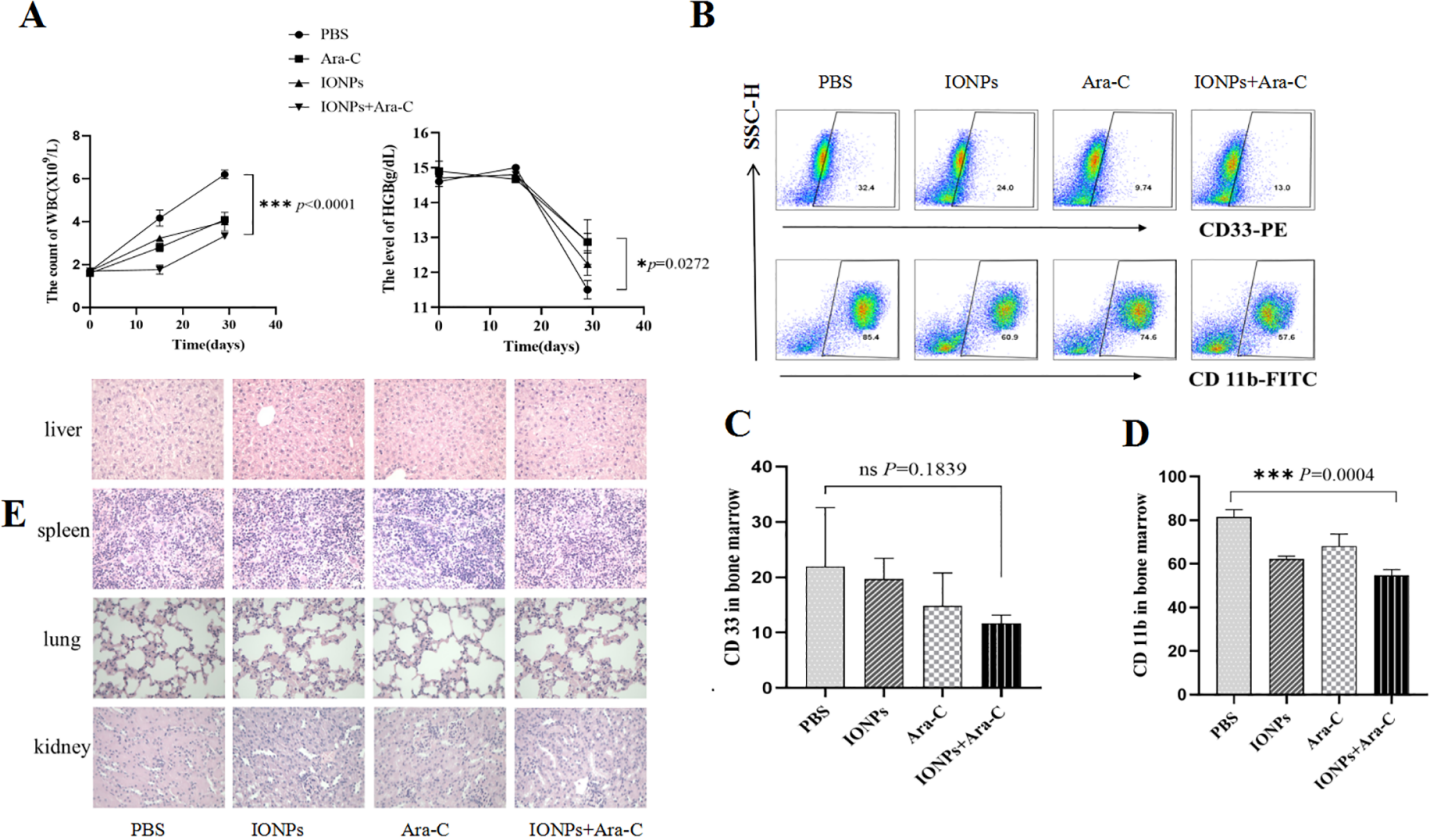
Evaluating the inhibitory effects of IONPs combined with Ara-C in AML model mice. **(A)** WBC counts and HGB levels in the peripheral blood from the treated mice. **(B)** The CD33 and CD11b expressions in the bone marrow cells as detected by FCM. **(C)** Statistical results of the CD33 and CD11b expression levels. **(D)** The H&E staining of the spleen, liver, kidney, and lung tissues (×400). *P < 0.05, ***P < 0.001. ns, no statistical significance.

Finally, therapeutic effects of IONPs combined with Ara-C on the AML model mice were investigated. Twelve NOD/SCID mice were randomly divided into 4 groups of 3 mice each. The AML model mice were established again as described above. The disease status of the mice was observed and recorded daily. Blood was collected from the posterior orbital venous plexus of the mice and sent for a blood routine examination on Day 15. On Day 20, the mice were administered the injection in the same four ways described in the experimental method. After 30 days of the treatment (once every 3 days, in total of 10 times), the *in vivo* experiment was ended and the mice were routinely killed. The peripheral blood and bone marrow cells were collected for staining. **Figure 7A** shows the decreased counts of white blood cells (WBC) and the increased levels of hemoglobin (HGB) in the treated mice compared with the PBS control mice, suggesting that the treatment of IONPs and Ara-C led to expected efficacy of alleviating AML. **Figures 7B–D** show that IONPs plus Ara-C could inhibit the expression levels of CD33 and CD11b in the bone marrow cells from the AML-bearing mice more efficiently than a single drug treatment. Subsequently, we wanted to know whether these drugs would produce toxic damage to major organs and conducted pathological examinations to assess changes in the spleen, liver, kidney, and lungs of the mice. The results of H&E staining showed the normal morphology of the examined spleen, liver, kidney, and lung tissues from each treatment group, indicating that these drugs did not produce tissue toxicity (**Figure 7D**).

## Discussion

To investigate IFITM3 as a critical target gene in AML pathogenesis, we established stable KG-1a cell lines (LV-shIFITM3) using lentiviral-mediated knockdown. *In vitro* experiments demonstrated that IFITM3 downregulation effectively suppressed cell proliferation, colony formation, and anti-apoptotic capacity in these cells. CCK-8 assays further revealed that iron oxide nanoparticles (IONPs) inhibited KG-1a proliferation by reducing IFITM3 expression.

Previous studies have shown that carbohydrate-coated superparamagnetic IONPs can reduce tumor burden in bone marrow and spleen of AML model mice, exhibiting superior therapeutic efficacy compared to standard chemotherapy (Ara-C)^18^. The IONPs used in our study represent a novel nanozyme capable of elevating intracellular ROS levels while offering excellent biocompatibility, minimal toxicity, and scalable production advantages^19^.

As a positive control, we employed Prussian blue nanoparticles (PBNPs), which exhibit multi-enzyme activities and have been widely applied in biomedical fields^20^. Notably, Bai et al. demonstrated their utility in targeted chemo-photothermal therapy for AML^21^. Our comparative analysis revealed that IONPs more effectively suppressed both IFITM3 expression and KG-1a proliferation than PBNPs.

Mechanistic studies indicated that IONPs downregulate IFITM3 expression, subsequently inhibiting c-Myc and NF-κB p65 phosphorylation- two key regulators of cell proliferation. This pathway likely contributes to the observed anti-proliferative effects in KG-1a cells.

Because Ara-C being is a conventional chemotherapy drug for AML, in this study, we employed IONPs in combination with Ara - C to evaluate how this treatment would enhance the suppression of AML. The results of the *in-vitro* experimental results demonstrated that the combination of IONPs and Ara-C was capable of inducing apoptosis in KG-1a cells and promoting the production of ROS. As the elevation of ROS is beneficial for the specific killing of AML cells^22–24^, this accounts for the significant increase the ROS levels in KG -1a cells co - incubated with IONPs and Ara - C, as opposed to those treated with Ara - C alone. This finding suggests that IONPs enhanced the sensitivity of KG -1a cells to Ara - C, which was closely linked to the augmented production of ROS.

To verify the *in-vitro* experimental outcomes, it is necessary to conduct an *in-vivo* experiment. Initially, we constructed an AML-bearing mouse model by inoculating KG -1a cells into NOD/SCID mice through their tail veins. Eight weeks later, the mice inoculated with KG -1a cells started to display symptoms such as lethargy and alopecia.

In comparison, the mice injected with PBS remained active, in good spirits, and had lustrous hair. Furthermore, only a small number of primitive and undifferentiated cells were observed in the bone - marrow smears of the PBS- injected mice, whereas leukemia cells were detected in the peripheral blood of the KG -1a- injected mice, along with a substantial number of primitive cells in their bone- marrow smears. These results confirmed the successful establishment of the AML mouse model for this study.

Subsequently, each mouse was inoculated with either 5×10^6^ Lv-shIFITM3-KG1a cells or scrambled control cells. Forty days post-inoculation, the harvested peripheral blood and bone marrow cells were stained and analyzed. Under the fluorescence microscope, due to the gene knockdown effect on IFITM3 expression, significantly fewer IFITM3-expressing cells were observed in the samples from mice injected with Lv-shIFITM3-KG1a cells compared to those injected with scrambled control cells. Moreover, the mice administered Lv-shIFITM3-KG1a cells exhibited enhanced mobility, better overall health status, and shinier fur, contrasting markedly with the mice injected with scrambled control cells. These findings also confirmed that the knockdown of the IFITM3 expression effectively inhibited the growth process of the AML bearing mice in our experiment, and this approach may provide a the translational potential in clinical AML treatment. However, to further elucidate the role of IFITM3 in tumorigenesis and metabolism, additional studies are needed to investigate how IFITM3, as a key scaffold protein of the PI3K-Akt signaling pathway, enhances PI3K signaling by binding to phosphatidylinositol (3,4,5)-trisphosphate (PIP3) and its impact on AML progression^25^.

Numerous studies have demonstrated that CD33 is a critical target antigen in acute myeloid leukemia (AML). However, therapeutic strategies targeting the myeloid marker CD33 in AML patients often lead to collateral damage of normal myeloid cells, thereby inducing significant toxicity^26,27^.

Additionally, research has shown that CD11b positivity is a strong predictor of poor prognosis in AML patients, establishing the expression level of CD11b as a valuable prognostic biomarker^28^. Similarly, CD123 has emerged as a pivotal marker for the diagnosis, prognosis, and treatment of several hematological malignancies, particularly AML^29^.

These findings underscore the rationale behind our decision to detect the expression levels of CD11b, CD33, and CD123 in bone marrow cells. The flow cytometry (FCM) results were consistent with our *in vitro* findings. Specifically, in mice injected with Lv-shIFITM3-KG1a cells, the downregulation of IFITM3 expression led to a significant reduction in the expression levels of AML-associated immunological markers CD11b, CD33, and CD123. This compelling evidence further validates that reducing IFITM3 expression in KG1a cells effectively suppresses the progression of AML in mouse models.

Both the *in vitro* and *in vivo* experimental results demonstrated that IFITM3 promotes KG-1a cell proliferation and inhibits apoptosis. To further explore the therapeutic potential of this finding, we evaluated the efficacy of IONPs combined with Ara-C in treating AML by monitoring WBC counts and hemoglobin levels in AML-bearing mice, along with investigating the underlying mechanisms of treatment^30^. Our findings revealed that, compared with PBS-treated controls, mice receiving IONPs, Ara-C, or IONPs+Ara-C exhibited a significant reduction in WBC counts and an increase in hemoglobin levels, with the IONPs+Ara-C combination showing the most pronounced therapeutic effect. Furthermore, flow cytometry analysis demonstrated that the expression levels of AML-associated immunophenotypic markers (CD33, CD123, and CD11b) in bone marrow naïve cells were markedly downregulated 30 days post-treatment with IONPs+Ara-C.

Additionally, histopathological examination (H&E staining) confirmed that the spleen, liver, kidney, and lung tissues from treated mice maintained normal morphological architecture, indicating no detectable drug-induced tissue toxicity.

## Conclusions

This study demonstrates that IFITM3 plays a critical role in KG-1a cell proliferation and survival, suggesting its potential as a therapeutic target for AML. Moreover, the combination of IONPs and Ara-C effectively suppressed AML progression and alleviated disease symptoms in AML-bearing mice. Mechanistically, this synergistic effect may be attributed to IONPs-induced elevation of intracellular ROS levels, which enhanced KG-1a cell apoptosis and sensitized them to Ara-C treatment. These findings highlight IONPs-Ara-C combination therapy as a promising novel strategy for AML treatment.

## Data Availability

The datasets presented in this study can be found in online repositories. The names of the repository/repositories and accession number(s) can be found in the article/supplementary material.
